# The overlooked role of the seminovaginal microbiota in infertility: a narrative mini review

**DOI:** 10.3389/fmed.2026.1784130

**Published:** 2026-05-22

**Authors:** Charlotte Storck-Thy, Karen Angeliki Krogfelt, Rie Jønsson

**Affiliations:** Department of Science and Environment, Roskilde University, Roskilde, Denmark

**Keywords:** fertility, infertility, microbiota, semen, vaginal

## Abstract

Infertility affects up to 15% of couples worldwide and is influenced by complex biological, immunological, and environmental factors. While reproductive microbiome research has expanded rapidly, the majority of published studies remain largely descriptive, focusing on taxonomic profiling rather than functional or mechanistic insight. Findings from vaginal microbiome studies have been heterogeneous and strongly influenced by methodological variation, and the male genital microbiome has received comparatively less attention, particularly in a couple context. This narrative mini review provides a synthesis of studies investigating paired male and female reproductive microbiomes, emphasizing observational evidence of partner- associated microbial patterns in relation to fertility outcomes. The term ‘seminovaginal’ microbiota is discussed as a hypothesis describing the transient and dynamic interface arising during sexual activity. Current evidence is limited, largely associative, and constrained by insufficient standardization of sampling, sample handling and processing, data analysis, restricting causal interpretation. Addressing these gaps through coupled, longitudinal, and mechanistic study designs is essential for advancing biologically meaningful conclusions in infertility research and reproductive treatments.

## Introduction

The female reproductive tract is a highly regulated mucosal environment, shaped by hormonal fluctuations, immune surveillance, and microbial colonization. The vaginal microbiome, typically dominated by *Lactobacillus* species, plays a protective role by maintaining low pH and producing antimicrobial metabolites such as lactic acid and bacteriocins (1). Dysbiosis, characterized by a shift toward facultative anaerobic bacteria such as *Gardnerella*, *Prevotella*, and *Atopobium*, has been linked to increased susceptibility to sexually transmitted infections (STIs), bacterial vaginosis (BV), and poor reproductive outcomes (2–4). Traditionally, the body’s microbiotas within a single person have been viewed separately, mainly thought to affect the closest organs. However, recent research has revealed that the vaginal microbiome is influenced by a broader systemic and behavioral factors such as menstrual cycle, choice of menstrual products, and ethnicity (5–7). These observations highlight the dynamic nature of the vaginal ecosystem and support the emerging hypothesis that microbial interactions may extend beyond individual anatomical sites, including between sexual partners.

## Search strategy and selection criteria

This mini review was conducted as a narrative synthesis of the literature on paired male and female genital microbiomes in fertility context. A structured search was performed in PubMed and Google Scholar between June and November 2025 to identify relevant studies. Although a substantial body of literature exits on vaginal microbiome and the seminal microbiome when studied independently, only a limited number of studies have exanimated these sites simultaneously within couples, and in the context of fertility or ART outcomes.

Two search strategies were used:

1. *“(((vaginal) AND (semen)) AND (microbiome)) AND (microbiota) + humans filter,”* yielding 42 records, and
2. *“(((vagina) AND (semen)) AND (infertility)) + humans filter,”* yielding 69 records.

After running the two search strategies, all records were exported and screened in a shared spreadsheet. Animal studies, non-human models and duplicates were removed. This resulted in a set of studies primarily focusing on human reproductive microbiota, including both narrative and systematic reviews, male-only or female-only studies, and couple-based designs.

We then screened full texts to identify studies that: (i) investigated heterosexual couples, (ii) included microbiome profiling of both semen and vaginal samples, and (iii) addressed fertility or ART outcomes. This yielded 11 studies that met these criteria. Of these three were excluded at the final stage: one was a study protocol without published microbiome results, one focused exclusively on sample handling and did not report microbiome composition, and one did not, upon closer inspection, present microbiome data despite initial indexing. Ultimately eight studies fulfilled all criteria and were included (see Table 1).

**Table 1:** Based on the eight studies included in this mini-review, the table provides an overview of study characteristics, including the number of infertile and control couples, sampling sites, analytical techniques for bacterial identification, as well as key findings and study limitations.

Study (ref. nr.)	Study design	Infertile couples	Control couples	Sampling sites	Analytical technique	Key findings	Limitations
Koort et al. (28)	Observational study, cross-sectional study	97	12	Vaginal secrete and semen	Illumina 16S rRNA sequencing, Nugent score, gram staining	Positive ART outcome correlates with vaginal *L.crispatus* or *Lactobacillus* sp. and *Acinetobactor* in semen microbiome.	Small control group. Not longitudinal. No data on sexual praxis.
Baud et al. (11)	Observational study, cross-sectional study	65	None	Vaginal swap, penis swap, semen and follicle fluid.	Illumina 16S rRNA sequencing, qPCR.	The vaginal microbiome is dominated by *Lactobacillus* and men have a higher microbial diversity.	No control group. Not longitudinal. No data on sexual praxis.
Mändar et al. (9)	Observational study, cross-sectional study	23	None	Vaginal swap and semen	Illumina 16S rRNA sequencing.	Semen microbiome is more diverse but has low biomass. Intercourse changes the vaginal microbiome. *G. vaginalis* for women correlates with leukocytospermia for men.	No control group. Small sample size. Not longitudinal.
Okwelogu et al. (23)	Observational study, cross-sectional study	36	None	Vaginal swap and semen	Illumina 16S rRNA sequencing	Positive IVF outcome correlates with a higher presence of *Lactobacillus jensenii* and *Faecalibacterium* in semen. Negative IVF outcome correlates with higher presence of *Prevotella, Bacteroides*, and lower *Firmicutes/Bacteroidetes-*ratio.	No control group. Small sample size. No data on sexual praxis. Not longitudinal.
Borovkova et al. (30)	Observational study, cross-sectional study	20	None	Vaginal swap and semen	Culturing, PCR, Nugent score, gram staining.	Intercourse results in a higher similarity between the male and female microbiota, especially transfer of anaerobic bacteria.	No control group. Small sample size. No data on sexual praxis. No microbiome sequencing.
Štšepetova et al. (32)	Observational study, cross-sectional study	50	None	Vaginal swap and semen	454 pyro-sequencing and qPCR	*Alphaproteobacteria* correlates with a positive embryo quality whereas *Bacteroidetes* correlates negatively with sperm motility.	No control group. Small sample size. No data on sexual praxis.
Huerga López et al. (18)	Randomized, triple-blind, placebo-controlled clinical trial	70	None	Vaginal swap and semen	Illumina 16S rRNA sequencing, qPCR, culturing, ELISA	Probiotics given to both partners increased successful pregnancies and improved immune profile in the women.	No control group. Small sample size. No data on sexual praxis.
Ricci et al. (29)	Prospective Cohort Study	285	None	Vaginal swap and semen	Culturing, qPCR, spermigram	IVF was successful in 85.7% of couples without detected genital pathogens, only 7.5% in couples harboring *E. faecalis, U. urealyticum*, and/or *M. hominis. E. faecalis* significantly reduced sperm motility and morphology.	No control group. Small sample size. No data on sexual praxis. No microbiome sequencing.

The data extraction focused on the study design, sample types, sequencing methods, microbial taxa, evidence suggestive of for the microbial exchange between partners, association with fertility or artificial reproductive treatment (ART) outcomes, and whether the menstrual cycle was considered. As summarized in Table 1, the included studies are characterized by small cohort sizes, limited use of control groups, heterogeneous analytical approaches, and largely descriptive designs, which constrain the interpretability of the available evidence.

## Reproductive microbiome context in fertility research

The vaginal microbiome is commonly discussed in relation to the endometrial environment, reflecting the microbial, metabolic, and immunological conditions in the lower reproductive tract may influence uterine receptivity and implantation. Studies of other reproductive niches, including the endometrium, shows that biologically relevant effects may arise even from low-biomass and potentially transient microbial or immunological signals (8).

## The seminovaginal microbiota: a hypothesis on microbial exchange between partners

In this mini review, we use the term seminovaginal microbiota as a hypothesis describing the transient or potentially short-lived microbial interface created during sexual activity as described by Mändar et al. (9). Importantly, this term does not imply the existence of, a stable or discrete biological ecosystem, but is used as a framework to describe microbial transfer and interaction between semen and the vaginal environment. Furthermore, existing studies do not evaluate persistence, temporal dynamics or functional integration, which potentially play a role in fertility. Recognizing that fertility related processes may not be adequately understood by examination either microbiome in isolation.

**Figure 1:**
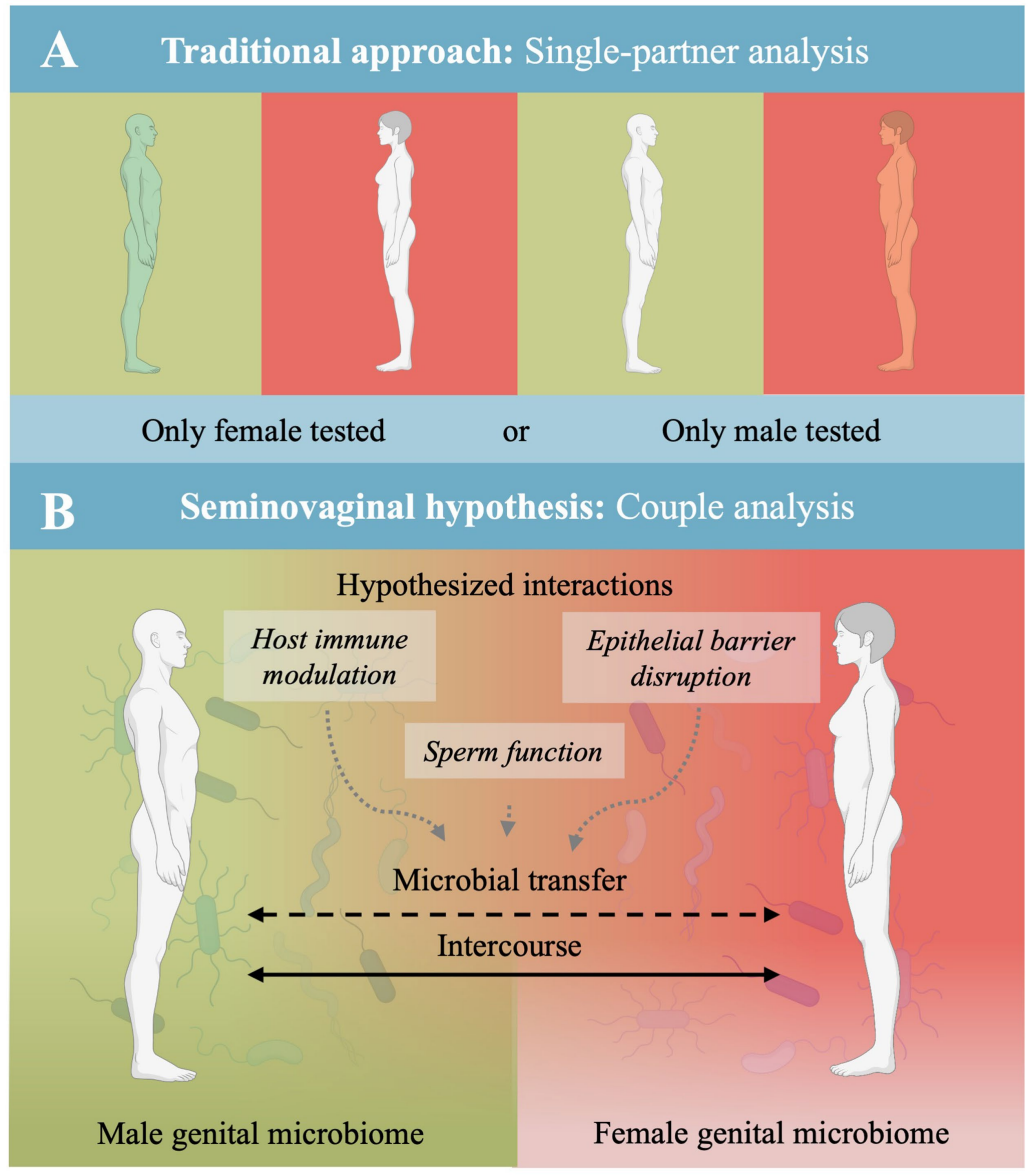
The seminovaginal microbiota hypothesis as an overlooked approach in infertility research. **(A)** Illustrates the traditional approach, in which male and female reproductive microbiomes are typically studied in isolation. **(B)** Presents the proposed seminovaginal hypothesis, highlighting a transient and dynamic microbial interface occurring during sexual intercourse. The figure does not imply a stable or discrete shared ecosystem but emphasizes a couple-level interactional context that is largely underexplored in infertility research. Made partly in biorender.com.

To illustrate the conceptual distinction between the prevailing single-partner analytical approach and a proposed couple-level perspective, Figure 1 contrasts traditional reproductive microbiome analyses with the seminovaginal hypothesis. As shown in Figure 1A, most existing studies focus on either the female or the male partner in isolation. In contrast, Figure 1B depicts the seminovaginal hypothesis as a transient, interactional interface arising during sexual intercourse, emphasizing hypothesized contact-dependent processes rather than stable microbial colonization.

Studies have shown that semen and vaginal microbiota share key taxa, and that intercourse is capable of altering the vaginal microbiota (9–11). Mändar et al. (9) showed that *Gardnerella vaginalis* abundance in women correlates with leukocytospermia in male partners, suggesting a potential association between partner associated microbiota and inflammatory markers. Baud et al. (11) found limited but detectable microbial transmission between partners, with genera such as *Prevotella*, *Staphylococcus*, and *Porphyromonas*, some of which are associated with bacterial vaginosis, present in both semen and vaginal samples. While these findings are consistent with microbial exchange between partners, they remain observational and do not establish a functional impact. Proposed downstream effects, such as epithelial barrier disruption, modulation of cytokine profiles, or effects on implantation are therefore hypothesis generating, rather than evidence-based conclusions.

It has previously been suggested to treat bacterial vaginosis (BV) as a sexually transmitted disease (12, 13). Last year a multicenter randomized trial published in the New England Journal of Medicine (10) demonstrated that treating both female and male partners with oral metronidazole and topical clindamycin significantly reduced BV recurrence rates compared to only females treated (35% vs. 63%). While these findings support an association between the male genital microbiota and vaginal microbial composition, the mechanism underlying this effect remain incompletely understood. Nonetheless, these findings illustrate that repeated, transient microbial exposure between partners may be biologically meaningful even when a stable microbial persistence has not been demonstrated. Such interactional effects are not captured by single-partner analysis and support the relevance of a couple-level perspective in reproductive medicine.

## Male genital microbiome and its effect on female reproductive health

The seminal microbiome is not sterile and contains a diverse array of bacteria, some of which have been associated with male infertility (14). An estimated 6–10% of infertility cases involve infections or inflammation in the male genital tract (15). Despite this, the male genital microbiome, particularly in semen and penile tissues, has received limited attention in studies investigating host-pathogen interactions. Emerging evidence suggests that microbial exchange between sexual partners, as well as dysbiosis in the male reproductive tract, may influence female reproductive health, immune responses, and fertility outcomes (9, 11, 16–19). However, most studies are observational, and causality has not been established.

Several studies have reported that semen dominated by *Lactobacilli* has been associated with high quality semen (17, 20, 21). Other taxa such as *Brevundimonas, Staphylococcus, Flavovacterium* and *Pelmonas* have been identified in semen samples with normal motility and low DNA fragmentation (17, 22–24). These associations are descriptive, and the functional implications of these taxa remain to be determined.

Grande et al. (16), Osadchiy et al. (25) and Garcia-Segura et al. (22) reported that dysbiosis in semen, characterized by the prevalence of genera such as *Prevotella*, *Finegoldia*, *Lactobacillus iners, Pseudomonas, Peptoniphilus*, and *Gardnerella*, is associated with oxidative stress, DNA fragmentation, and reduced sperm motility. Several of these genera have also linked to responsible for dysbiosis in the female reproductive tract, including bacterial vaginosis (BV), although whether they play similar mechanistic roles in both partners remain unclear.

*In vitro* studies have shown that BV-associated molecules such as vaginolysin and lipopolysaccharides (LPS) from *Gardnerella* and *Prevotella* are capable of impairing sperm capacitation and acrosome reaction (26). These findings suggest potential mechanisms, but their relevance *in vivo* has not yet been fully established. Microbial metabolites such as short-chain fatty acids (SCFAs) and polyamines have been proposed to modulate host immunity and hormone levels, which could influence reproductive outcomes (27).

## Impact on ART and clinical outcomes

Microbiome compositions in both partners have been associated with variations in ART outcomes in observational studies (28, 29). Evidence from observational studies suggest that *Lactobacillus crispatus* dominance in the vagina and semen is associated with higher implantation and live birth rates (18, 30, 31). The recovery of *L. crispatus* from embryo transfer catheters has been explored as a potential prognostic indicator (28). Contrary, BV-associated taxa such as *Gardnerella*, *Atopobium*, and *Prevotella* have been associated with implantation failure and miscarriage (31, 32). These associations do not establish a causality but point toward a potential pathway and highlight the need for mechanistic and interventional studies in ART.

## Menstrual cycle and microbiome dynamics

The vaginal microbiome is highly sensitive to hormonal changes across the menstrual cycle (33). During menstruation, the acidic environment is neutralized, favoring anaerobic bacteria like *Gardnerella*, *Streptococcus*, and *Escherichia coli* (34). Hugerth et al. (6) introduced the concept of vaginal community dynamics (VCDs). This study showed that sexual activity and menstruation can trigger transitions between eubiotic and dysbiotic states in the vaginal tract, for some of the females, while other females were constant during a full cycle. These findings underscore the importance of considering both partners in microbiome studies and controlling the timing of sample collection.

Sampling without accounting for cycle phase may lead to misclassification and confounded results. Despite this, many studies fail to report or control for menstrual phase, limiting reproducibility and clinical relevance (4, 28).

## Methodological challenges and recommendations

Despite promising findings, several methodological challenges remain for the existing studies. One major limitation is the low biomass and high risk of contamination, particularly in semen and follicular fluid samples (13). These sample types often contain small amounts of bacterial DNA, making them vulnerable to contamination. When processing these sample types, even minimal contaminations can affect sequencing and thereby masking the true biological relevance. Another challenge is the high variability of sequencing methods and bioinformatic processes, a lack of standardization that undermines the robustness and comparability of the studies.

Another challenge is the insufficient control of the menstrual cycle, despite the well documented hormonal effects on immune function and microbiome stability (28, 35). Many studies collect samples at a single, uncontrolled time point, making it difficult to determine the origin of the observed differences. In addition, most studies include only the female or male partner, thereby overlooking the role of the microbial exchange between partners.

Finally, as evident from Table 1, most studies are cross-sectional studies, which means that the temporal dynamics, microbial persistence and effect of intercourse are rarely captured. Without these longitudinal biological samples, it is challenging to identify the causality or relevance of the role of the microbiota.

To address these gaps, future research should include negative and low biomass controls, standardize sequencing and analysis pipelines, include paired sampling from both partners. As conceptually illustrated in Figure 1, the widespread reliance on single-partner sampling limits the ability to capture interactional and contextual processes that may be relevant for fertility-related outcomes. In addition, studies should control for menstrual cycle phase and hormone status, assess possible antibiotics treatment for both partners, and furthermore include multi-omics approaches to gain functional insights, and employ longitudinal designs to monitor microbial shifts over time.

## Future directions and interventions

Emerging strategies aim to modulate the reproductive microbiome in ways that may support fertility, although most evidence remains preliminary. Intervention studies such as probiotics and postbiotics have shown promising but not definitive effects (36). For example, the oral administration of *Ligilactobacillus salivarius* has been associated with improved pregnancy rates and immune profiles in couples with unexplained infertility (18), but the underlying mechanisms and generalizability of these findings remain to be established.

Microbiome screening before ART, combined with emerging predictive algorithms based on vaginal and seminal microbiota has been proposed as an approach to inform future research and patient stratification, rather than current clinical decision making (3). However, these approaches are still experimental and require validation in larger, prospective cohorts before clinical implementation can be recommended.

The integration of multiomics, combining genomics, transcriptomics, proteomics, metabolomics and immune profiling, may provide deeper mechanistic insights into host–microbe interactions within and between partners and help clarify whether the observed associations reflect causal pathways or microbial variations (37). Such methods, particularly when applied longitudinally, may also reveal patterns of microbial resilience, partner specific microbial taxa exchange, and temporal dynamics that cannot be captured through standard sequencing alone.

## Conclusion

The male genital microbiome appears to be potentially relevant as it has been shown to correlate with reproductive outcomes, particularly in the context of interaction with the female reproductive tract. However, the limitations summarized in Table 1 shows that much of the current evidence is associative, derived from small cohorts, or based on *in vitro* work. To better understand the potential role of microbial exchange and male-derived dysbiosis in infertility, future studies should integrate male microbiome profiling alongside female sampling, account for menstrual cycle timing, incorporate standardized sequencing and analytical methods. Such efforts will be essential for determining whether the hypothesis of the seminovaginal microbial interface reflects a biologically meaningful association with infertility or primarily serves as a marker of underlying reproductive health.
